# Phase 1 drug-drug interaction study to assess the effect of CYP3A4 inhibition and pan-CYP induction on the pharmacokinetics and safety of fosmanogepix in healthy participants

**DOI:** 10.1128/aac.01650-23

**Published:** 2024-05-17

**Authors:** Michael R. Hodges, Sjoerd van Marle, William G. Kramer, Eric Ople, Margaret Tawadrous, Abhijeet Jakate

**Affiliations:** 1 Independent / Former Amplyx and Pfizer, San Diego, California, USA; 2 ICON, Groningen, the Netherlands; 3 Kramer Consulting LLC, North Potomac, Maryland, USA; 4 Independent / Former Amplyx Pharmaceuticals, Inc., San Diego, California, USA; 5 Pfizer Inc., Peapack, New Jersey, USA; University of Iowa, Iowa City, Iowa, USA

**Keywords:** CYP450, itraconazole, rifampin, FMGX/APX001, MGX/APX001A

## Abstract

Immunocompromised patients are susceptible to fungal infections, and drug-drug interactions with antifungals may occur due to concomitant medications. Fosmanogepix [FMGX; active moiety manogepix (MGX)] targets glycosylphosphatidylinositol-anchored mannoprotein synthesis and maturation, essential for fungal virulence. This phase 1, fixed-sequence study in healthy participants evaluated the effect of strong CYP3A4 inhibitor itraconazole [Cohort 1 (*n* = 18); FMGX 500 mg intravenous (IV) twice a day (BID )+ itraconazole 200 mg oral once a day (QD)] and pan-CYP inducer rifampin [Cohort 2 (*n* = 18); FMGX 1,000 mg IV BID + rifampin 600 mg oral QD] on the pharmacokinetics of FMGX and MGX. In cohort 1, geometric mean (GM) MGX C_max_, AUC_0-t_, and AUC_inf_ were almost similar with and without itraconazole administration. In Cohort 2, GM MGX C_max_ was slightly lower and AUC_0-t_ and AUC_inf_ were significantly lower after rifampin administration, with the least squares GM ratio associated 90% confidence intervals (CIs) below 80 – 125% (no effect window). No deaths, serious adverse events (SAEs), or FMGX-related withdrawals were reported. In both cohorts, a total of 188 AEs (*n* = 30; 186 mild; two moderate) were reported. In all, 37 of 188 AEs (*n* = 12) were considered FMGX related (most frequent: headache, nausea, and hot flush). Administration of FMGX alone and with itraconazole or rifampin was safe and well tolerated. A strong CYP3A4 inhibitor had no effect on FMGX or MGX exposure. A strong pan-CYP inducer had no effect on FMGX exposure but demonstrated ~45% decrease in MGX exposure.

**CLINICAL TRIALS:**

This study is registered with ClinicalTrials.gov as NCT04166669 and with EudraCT as number 2019-003586-17.

## INTRODUCTION

Invasive fungal diseases (IFDs) affect more than 800 million people worldwide and are associated with 1.66 million deaths per year (1). IFDs are commonly linked to immunosuppressive conditions, for which patients receive concomitant medications that may increase the risk of drug-drug interactions (DDIs) with antifungals (2).

Cytochrome P450 (CYP) enzymes are primarily found in the liver and are responsible for the metabolism of ~75% of drugs, 30% of which are metabolized by CYP3A4 and CYP3A5 enzymes. CYPs can be inhibited or induced by other drugs, thus influencing treatment outcomes through DDIs (3). Currently used antifungals have a diverse CYP metabolism profile. Amphotericin B (a polyene) has broad activity against yeasts and molds with no known CYP-related DDIs but with limited use due to renal toxicity. Triazoles are commonly used for prophylaxis and to treat fungal infections in immunocompromised patients but show significant DDIs through CYP inhibition or membrane transporters (2, 4). Among the echinocandins, caspofungin is not a CYP inhibitor but shows some DDIs when administered with cyclosporine A, tacrolimus, and enzyme inducers. Anidulafungin and micafungin have a low DDI potential warranting no dose adjustment with co-administered medications (2).

Fosmanogepix (FMGX), the first member of the “gepix” class of antifungals (5), is a N-phosphonooxymethyl prodrug of manogepix (MGX) (6). After oral or intravenous (IV) administration, it is rapidly metabolized to its active form MGX by systemic phosphatases (6). MGX acts by inhibiting the fungal enzyme glycosylphosphatidylinositol-anchored wall transfer protein (GWT1) that leads to disruption of glycosylphosphatidylinositol (GPI)-anchored protein synthesis and maturation of mannoproteins. Mannoproteins are involved in fungal cell wall synthesis, organization and re-modeling, and virulence. In addition, the closest mammalian ortholog of GWT1, phosphatidylinositol glycan anchor biosynthesis class W (PIGW), is not inhibited by MGX (5, 6). FMGX has demonstrated efficacy in various mouse models of invasive pulmonary and disseminated fungal infections caused by *Candida*, *Coccidioides*, *Cryptococcus*, and *Aspergillus* spp (7). In mice infected with *C. albicans* and *C. glabrata* (including echinocandin-resistant and multidrug-resistant isolates), pre-administration of a nonselective CYP inhibitor, 1-aminobenzotriazole, was found to increase the exposure [area under the concentration-time curve (AUC)] and half-life of MGX (8).

The metabolite patterns of MGX were assessed *in vitro* after incubation with mouse, rat, dog, monkey, and human cryopreserved hepatocytes, and human liver microsomes. The results indicated that multiple recombinant human CYP (rCYP) enzymes were involved in the formation of MGX-derived metabolites, including rCYP1A2, rCYP2A6, rCYP2B6, rCYP2C9, rCYP2C19, rCYP2D6, rCYP2E1, rCYP2J2, rCYP3A4, and rCYP3A5 (data on file).

No reversible or time-dependent inhibition of human CYPs at concentrations up to 30 µM *in vitro* was observed for FMGX. Reversible inhibition potency of MGX was observed for CYP1A2, CYP2C9, CYP2D6, CYP3A4, CYP2B6, CYP2C8, and CYP2C19. Weak or no inhibition was observed for other CYP isoforms. MGX demonstrated no time-dependent inhibition of CYP1A2, CYP2A6, CYP2C8, CYP2C9, CYP2C19, CYP2D6, and CYP2E1, and weak time-dependent inhibition of CYP2B6 and CYP3A4 (nifedipine CYP3A4 substrate). In addition, mechanistic modeling of the area under the curve ratio (AUCR) values for oral and IV routes was conducted for MGX using a mean maximum concentration (C_max_) of 20.0 µg/mL, the highest observed in the Phase 1 clinical trials. For IV administration, the only AUCR value exceeding 1.25 was noted for CYP3A4 and for oral administration, AUCR values exceeding 1.25 were noted for CYP2C8, CYP2D6, and CYP3A4 (data on file).

Previously, in a Phase 1 oral study of FMGX in healthy volunteers (NCT02957929) (9), the DDI potential of FMGX as a perpetrator was studied by assessing its effect on the Geneva cocktail of CYP substrates, with six unique probe substrates primarily metabolized by CYPs 1A2, 2B6, 2C9, 2C19, 2D6, and 3A4, respectively (10) administered alone and after dosing of FMGX 500 mg once daily (QD) for 14 days. However, the effect of CYP induction or inhibition on FMGX or MGX metabolism has not yet been studied.

The concomitant administration of FMGX with multiple medications that are strong CYP inhibitors may increase MGX exposure levels (as a victim substrate) and may negatively impact the safety and tolerability of FMGX or conversely, the concomitant administration of medications that are strong CYP inducers may decrease MGX levels (as a victim substrate) and may reduce the clinical efficacy of FMGX.

This study was conducted to evaluate the effect of a strong CYP3A4 inhibitor (itraconazole) and a pan-CYP inducer (rifampin) on the single-dose pharmacokinetics (PK) of MGX and FMGX in healthy participants. Itraconazole and rifampin (including dose, dosage form, and treatment duration) were chosen as the CYP3A4 inhibitor/CYP inducer based on Food and Drug Administration (FDA) recommendations for DDI studies (11–13). The results of this study will provide insights into concomitant medication use during FMGX treatment.

## MATERIALS AND METHODS

### Study design and participants

In this open-label, fixed-sequence, phase 1 DDI study (NCT04166669; EudraCT number: 2019-003586-17), healthy adult participants [aged 18–60 years; body mass index (BMI): 18.0–32.0 kg/m^2^; weight ≥50 kg) were included. Key exclusion criteria were administration of any drug/herbal CYP3A modulator (e.g., erythromycin, St. John’s Wort) within 4 weeks or five half-lives (whichever was longer) or any other nutrients that can act as CYP3A modulators (e.g., grapefruit juice; Seville orange) within 2 weeks prior to the first admission, history of clinically significant allergic drug reactions, prior FMGX exposure, and known hypersensitivity to itraconazole or rifampin. Participants were also excluded if they used any prescription medication (except female contraception) and any over-the-counter medications/supplements within 14 days and 7 days prior to the first admission, respectively. Participants were to refrain from consuming foods containing poppy seeds and performing strenuous exercise, 48 and 96 hours prior to screening and first admission, respectively. In addition, in the cohort evaluating the effect of a pan-CYP inducer (rifampin), participants were excluded if they or their first-degree relatives had a diagnosis or were suspected to have porphyria, or if they were unwilling to abstain from using contact lenses during rifampin dosing.

### Treatments

**Fig 1:**
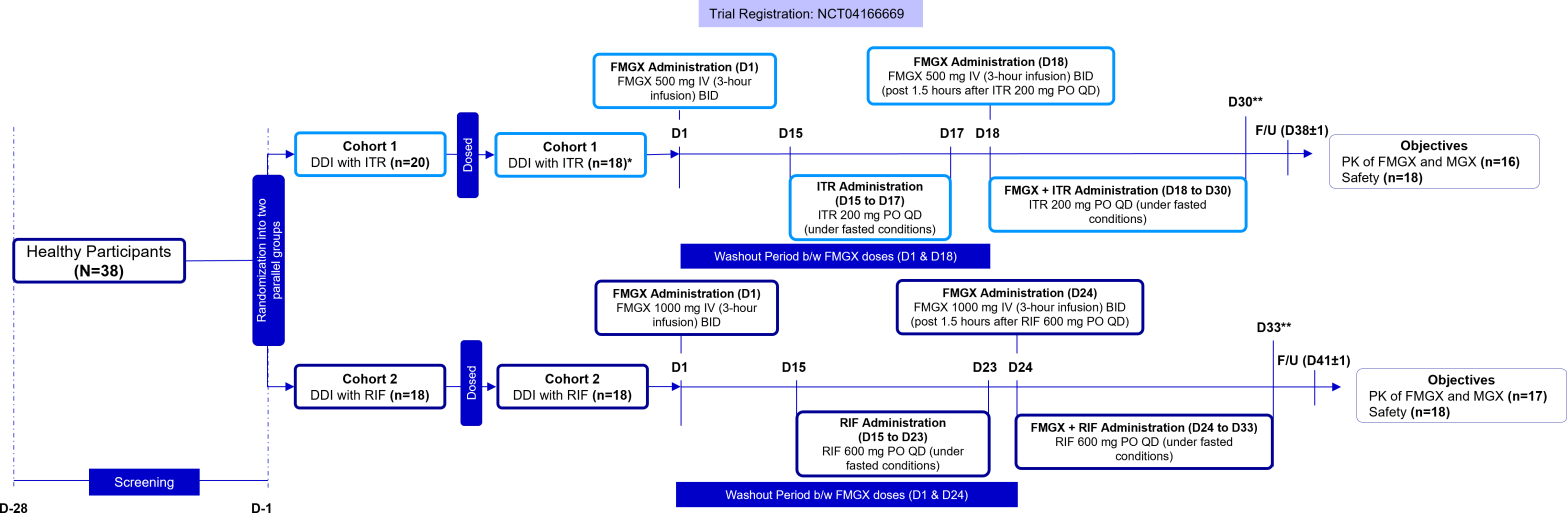
Study design. *, Two participants were randomized but withdrew consent before dosing; **, participants were discharged on D31 (Cohort 1) and D34 (Cohort 2). B/W, between; BID, twice a day; D, day; DDI, drug-drug interaction; FMGX, fosmanogepix; F/U, follow-up; ITR, itraconazole; IV, intravenous; MGX, manogepix; PK, pharmacokinetics; PO, oral; QD, once daily; RIF, rifampin.

Participants were grouped into two parallel cohorts: Cohort 1 (DDI with itraconazole) and Cohort 2 (DDI with rifampin). In both cohorts, FMGX was administered as a 3-hour IV infusion twice a day (BID, dosing interval: ~9 hours). All participants received FMGX on 2 days, once before a washout period (minimum 14 days) and once after the washout. Itraconazole/rifampin administration began 14 days after the first dose of FMGX and was ongoing during the second administration of FMGX. Cohort 1 received FMGX 500 mg on day 1, oral itraconazole 200 mg QD (12) under fasted conditions from day 15 to 30, and FMGX 500 mg again on day 18 (1.5 hours after itraconazole dosing). Cohort 2 received FMGX 1,000 mg on day 1, oral rifampin 600 mg QD (11, 13) under fasted conditions from days 15 to 33, and FMGX 1,000 mg again on day 24 (1.5 hours after rifampin dosing) (Fig. 1). The study consisted of a screening phase [days −28 to −1 (admission)], assessment phase (cohort 1: days −1 to 31; cohort 2: days −1 to 34), and follow-up phase (cohort 1: day 38 ± 1; cohort 2: day 41 ± 1). Participants in both cohorts had in-clinic stays for two periods: cohort 1 [days −1 to 4 (morning) and days 17 to 31 (morning)], cohort 2 [days −1 to 4 (morning) and day 23 to 34 (morning)], and at home dosing for two periods: cohort 1 (days 15 to 17) and cohort 2 (days 15 to 23).

Ambulatory visits were scheduled once every 2 days from days 6–14 and on days 15 and 16 (cohort 1) and once every 2 days from days 6–14 and on days 15 and 19 (cohort 2). Participants were discharged on day 31 (cohort 1) and day 34 (cohort 2).

### Assessments

The primary objective was to assess the effect of multiple doses of itraconazole (CYP3A4 inhibitor) and rifampin (pan-CYP inducer) on the PK of MGX and FMGX. In cohort 1, MGX plasma samples were collected at specified time points daily on days 1–4, once every 2 days on days 6–14, daily for 3 days from days 18 to 20, once every 2 days from days 21 to 29, and then on day 31, while itraconazole samples were collected at specified time points on days 19, 25, and 31. In cohort 2, MGX plasma samples were collected at specified time points daily on days 1–4, once every 2 days on days 6–14, daily for 3 days from days 24 to 26, once every 2 days from days 27 to 33, and then on day 34, while rifampin samples were collected at specified time points on days 25, 29, and 34. PK parameters were calculated using noncompartmental analysis from the plasma concentration-time data for MGX and FMGX. Primary PK parameters were maximum observed plasma concentration (C_max_), AUC from time 0 to infinity (AUC_0-inf_), and AUC up to time t (AUC_0-t_, where t was the last time point with concentrations above the lower limit of quantitation). Least squares geometric mean ratios [LSGMRs (90% confidence intervals (*CIs*))] were derived for comparisons of primary PK parameters for MGX with FMGX + itraconazole (day 18) or rifampin (day 24) versus (vs) FMGX alone (day 1). Secondary PK parameters were time to attain C_max_ (T_max_), AUC from time 0 to 23 hours (AUC_0-23_), terminal elimination half-life after the last dosing on the day (t_1/2_), clearance (CL), volume of distribution at terminal phase (Vz), and terminal phase rate constant after the last dosing on the day (λz).

The secondary objective was to assess the safety and tolerability of FMGX when administered alone and when co-administered with itraconazole or rifampin. Safety was assessed by evaluating the frequency of adverse events (AEs) recorded from the first admission (day −1) until completion of the follow-up visit (day 38 ± 1 in cohort 1; day 41 ± 1 in cohort 2). All AEs were classified per the Medical Dictionary for Regulatory Activities (MedDRA v22.1). The investigator ascertained relatedness to study the drug administration.

### Statistical methods, sample size, and analysis sets

An analysis of variance (ANOVA) model was used to analyze the natural log-transformed primary PK parameters while the secondary PK parameters were not subjected to inferential statistical analysis. For each cohort, 18 participants were included to ensure at least 16 completed all assessments, as this was considered adequate to evaluate PK, safety, and tolerability. Thus, a sample size of 36 was considered sufficient to assess primary and secondary objectives. The safety analysis set included all participants who received at least one dose of FMGX, itraconazole, or rifampin. The PK analysis set included all participants who received at least one dose of FMGX and had adequate plasma concentration data available for PK analysis.

## RESULTS

Of the 80 participants screened, 38 were randomized into two cohorts [cohort 1: number of participants (n)=20 and cohort 2: *n* = 18]. Of these, two participants in cohort 1 were randomized but withdrew consent prior to study drug administration, and 36 participants received the study drug (18 per cohort) and were included in the safety analysis set. A total of four participants who received study drugs discontinued the study (two per cohort). In cohort 1, the participants discontinued due to an AE (*n* = 1; mild anxiety) and personal reasons (*n* = 1) while in cohort 2, the reasons for discontinuations were family circumstances (*n* = 1) and voluntary withdrawal (*n* = 1). All four discontinuations were considered unrelated to any drug administration or study-related procedures. A total of 32 participants completed the study (16 in each cohort). However, one participant who discontinued (cohort 2) provided sufficient samples for PK analysis and the PK analysis set included 33 participants (cohort 1: *n* = 16; cohort 2: *n* = 17). Participants in both cohorts had comparable demographic characteristics with the majority being white males aged [mean (SD)] 43 years (14) and 41 years (12) in cohorts 1 and 2, respectively (Table 1).

**TABLE 1:** Baseline and demographic characteristics (safety analysis set)

Baseline characteristics	Cohort 1: DDI with ITR (*n* = 18)	Cohort 2: DDI with RIF (*n* = 18)
Age (years), mean (SD)	43 (14)	41 (12)
Gender, n (%)		
Female	6 (33.3)	7 (38.9)
Male	12 (66.7)	11 (61.1)
Race, n (%)		
White	16 (88.9)	15 (83.3)
Asian	0	1 (5.6)
White + Asian	2 (11.1)	2 (11.1)
BMI (kg/m^2^), mean (SD)	26.0 (2.8)	26.6 (4.3)

### Primary objective: PK of MGX and FMGX

**Fig 2:**
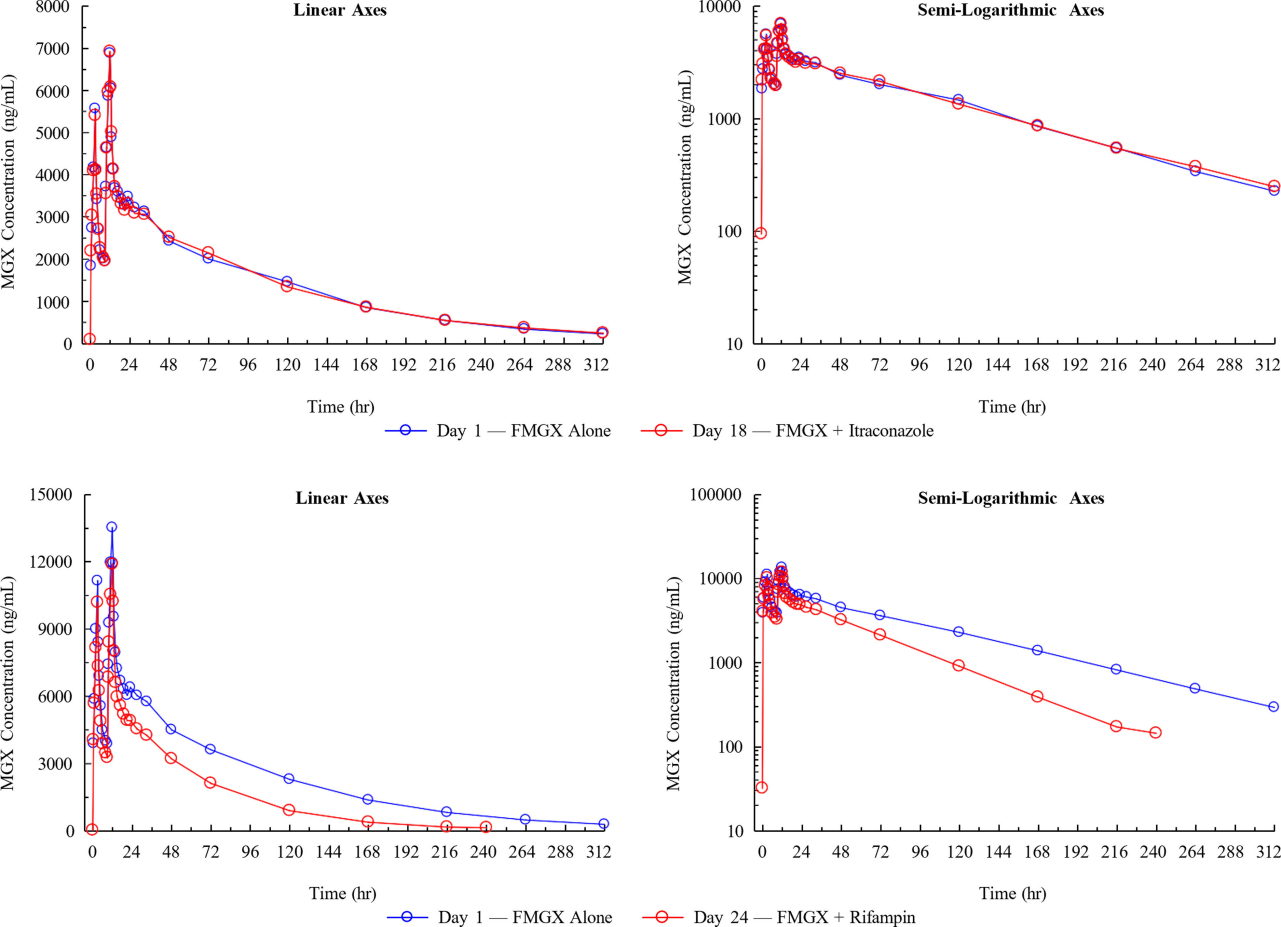
Geometric mean plasma concentrations of MGX: linear and semi-logarithmic axes. Cohort 1 (top panel): DDI with ITR [500 mg FMGX IV (3 hours infusion) BID alone and with ITR 200 mg oral QD × 16 days]. Cohort 2 (bottom panel): DDI with RIF [1,000 mg FMGX IV (3 hours infusion) BID alone and With RIF 600 mg oral QD × 19 days]. BID, twice a day; DDI, drug-drug interaction; FMGX, fosmanogepix; hr, hour; ITR, itraconazole; IV, intravenous; MGX, manogepix; QD, once daily; RIF, rifampin; MGX, manogepix.

In cohort 1, the geometric mean (GM) MGX plasma concentrations (post-FMGX administration) were superimposable before and after administration of itraconazole (Fig. 2). FMGX vs FMGX + itraconazole resulted in similar MGX GM values for C_max_ (6936 vs 7063 ng/mL), AUC_0-t_ (428571 vs 421057 h∙ng/mL), and AUC_inf_ (460142 vs 458447 h∙ng/mL) (Table 2). LSGMRs (90% CI) were 101.83% (97.00–106.91) for C_max_, 98.25% (95.26–101.33) for AUC_0-t_, and 99.63% (95.76–103.67) for AUC_inf_, indicating no effect of CYP3A4 inhibition on exposure to MGX (Table 3). In cohort 2 however, the GM MGX plasma concentrations were lower after administration of rifampin (Fig. 2). Administration of FMGX + rifampin vs FMGX alone resulted in slightly lower MGX GM C_max_ (12,112 vs 13,897 ng/mL) and significantly lower MGX GM AUC_0-t_ (435,791 vs 772,242 h.ng/mL) and AUC_inf_ (449,723 vs 8,219,708 h.ng/mL). The decrease in AUC was due to the increase in CL after induction of CYPs by rifampin (1,862 vs 3,403 mL/h; Table 2). For C_max_, the LSGMR (90% CI) was 87.16% (83.14–91.37), indicating no significant effect. However, for AUC_0-t_ and AUC_inf_, the LSGMRs were 56.43% (53.97–59.00) and 54.72% (52.26–57.29), respectively, demonstrating a 44-45% reduction in exposure to MGX by pan-CYP induction (Table 3).

**TABLE 2:** Summary of PK parameters for MGX (PK analysis set)^*b*^

	Cohort 1: DDI with ITR (*n* = 16)		Cohort 2: DDI with RIF (*n* = 17)	
PK parameter^*a*^	FMGX only (500 mg BID IV;D1)	FMGX (500 mg BID IV;D18) +ITR (200 mg QD PO; D15-30)	FMGX only (1000 mg BID IV;D1)	FMGX (1,000 mg BID IV;D24) +RIF (600 mg QD PO; D15–33)
C_max_ (ng/mL)	6,936 (23.2)	7,063 (19.4)	13,897 (31.0)	12,112 (31.8)
T_max_ (h)	12.0 (2.98–12.5)	12.0 (11.0–12.2)	12.0 (2.98–12.5)	12.0 (2.98–12.1)
AUC_(0-23)_ (h × ng/mL)	83,663 (23.2)	83,458 (21.2)	165,095 (32.6)	142,083 (31.1)
AUC_(0-t)_ (h × ng/mL)	428,571 (19.9)	421,057 (21.0)	772,242 (19.2)	435,791 (23.6)
AUC_(0-inf)_ (h × ng/mL)	460,142 (21.3)	458,447 (23.6)	821,908 (18.5)	449,723 (23.8)
t_1/2_ (h)	77.3 (25.4)	81.1 (28.2)	70.7 (36.1)	41.7 (33.6)
CL (mL/h)	1,663 (21.3)	1,669 (23.6)	1,862 (18.5)	3,403 (23.8)
Vz (L)	185 (25.1)	195 (22.4)	190 (40.8)	205 (42.4)

^*a*^ Geometric mean (geometric %CV) except for Tlag and Tmax for which the median (Range) is reported.

^*b*^ AUC_(0-23)_, area under the plasma concentration-time curve (AUC) from time 0 to 23 hours; AUC_(0-inf)_, AUC from time 0 to infinity; AUC_(0-t)_, AUC up to time t, where t = last point with concentrations above the lower limit of quantitation (LLOQ); C_max_, maximum observed plasma concentration; CL, clearance, calculated as dose/AUC_0-inf_; CV, coefficient of variation; D, day; PK, pharmacokinetic(s); PO, oral; QD, once daily; T_max_, time to attain C_max_; t_1/2_, terminal elimination phase half-life after the last dosing on the day; Vz, volume of distribution at terminal phase.

**TABLE 3:** Statistical comparison of PK parameters for MGX (PK analysis set)^*b*^^,^^*c*^

	Cohort 1: DDI with ITR (*n* = 16)			Cohort 2: DDI with RIF (*n* = 17)		
PK parameter	Least squares geometric means		Geometric mean ratio, % (90% CI)	Least squares geometric means		Geometric mean ratio, % (90%CI)
	FMGX only (500 mg BID IV;D1)	FMGX (500 mg BID IV;D18) + ITR (200 mg QD PO; D15–30)		FMGX only (1,000 mg BID IV;D1)	FMGX (1,000 mg BID IV;D24) + RIF (600 mg QD PO; D15–33)	
C_max_	6,936.12	7,063.21	101.83 (97.00–106.91)^*a*^	13,897.27	12,112.38	87.16 (83.14–91.37)
AUC_(0-t)_	428,570.72	421,057.07	98.25 (95.26–101.33)^*a*^	772,242.49	435,791.19	56.43 (53.97–59.00)
AUC_(0-inf)_	460,151.04	458,464.08	99.63 (95.76–103.67)^*a*^	821,907.66	449,722.78	54.72 (52.26–57.29)

^*a*^ The 90% CIs were within the 80%–125% no effect window.

^*b*^ Based on analysis of natural log-transformed data.

^*c*^ AUC_0-inf_, area under the plasma concentration-time curve (AUC) from time 0 to infinity; AUC_0-t_, AUC up to time t, where t = last point with concentrations above the lower limit of quantitation (LLOQ); BID, twice a day; C_max_, maximum observed plasma concentration; CI, confidence interval; D, day; DDI, drug-drug interaction; FMGX, fosmanogepix; ITR, itraconazole; IV, intravenous; MGX, manogepix; PK, pharmacokinetic(s); PO, oral; QD, once daily; RIF, rifampin.

In both cohorts, the GM FMGX plasma concentrations were comparable before and after administration of itraconazole or rifampin, with almost similar FMGX GM C_max_, AUC_0-t_, and AUC_inf_ (Table S1). The 90% *CIs* associated with the FMGX LSGMRs were also within a range of 80.00% to 125.00%, indicating that CYP3A4 inhibition or pan-CYP induction did not affect exposure to FMGX (data not presented). This is not unexpected since FMGX is not a substrate for CYP450 enzymes.

### Secondary objective: safety

No deaths, serious AEs (SAEs), or FMGX-related withdrawals were reported during the study. One participant from cohort 1 withdrew consent due to mild anxiety after receiving FMGX and itraconazole on day 18, not considered related to FMGX (Table 4). A total of 188 AEs were reported in 30 (83.3%) participants across the different treatment groups in both cohorts. The treatment groups in cohort 1 were FMGX only [AEs (E/n (%)): 21/9 (50.0)], itraconazole only [2/1 (5.9)], and FMGX + itraconazole [20/9 (52.9)]. The treatment groups in cohort 2 were FMGX only [49/14 (77.8)], rifampin only [47/13 (72.2)], and FMGX + rifampin [49/14 (82.4)]. Of all 188 events, 37 AEs reported by 12 (33.3%) participants were considered related to FMGX, with the number of AEs higher in cohort 2 (33/145 events) vs cohort 1 (4/43 events), possibly due to the higher dose of FMGX in cohort 2 (1,000 mg) compared to cohort 1 (500 mg). The most frequently reported AEs [E/n (%)] considered FMGX-related by the principal investigator were headache [7/6 (16.7)], nausea [6/4 (11.1)], and hot flush [6/6 (16.7) (Table 4).

**TABLE 4:** Overall safety summary with most frequent AEs by PT and summary of FMGX-related AEs by SOC and PT (safety analysis set)^*b*^^,^^*c*^

	Cohort 1: DDI with ITR (*n* = 18)			Cohort 2: DDI with RIF (*n* = 18)			Total (*N* = 36)
Safety parameters	FMGX only (500 mg BID IV; D1) (*n* = 18) E/n (%)	ITR only (200 mg QD PO; D15-17) (*n* = 17) E/n (%)	FMGX (500 mg BID IV; D18) + ITR (200 mg QD PO; D18–30) (*n* = 17) E/n (%)	FMGX only (1,000 mg BID IV; D1) (*n* = 18) E/n (%)	RIF only (600 mg QD PO; D15–23) (*n* = 18) E/n (%)	FMGX (1000 mg BID IV;D24) + RIF (600 mg QD PO; D24–33) (*n* = 17) E/n (%)	Overall E/n (%)
Any AEs	21/9 (50.0)	2/1 (5.9)	20/9 (52.9)	49/14 (77.8)	47/13 (72.2)	49/14 (82.4)	188/30 (83.3)
AEs by severity							
Mild	21/9 (50.0)	2/1 (5.9)	19/8 (47.1)	48/13 (72.2)	47/13 (72.2)	49/14 (82.4)	186/28 (77.8)
Moderate			1/1 (5.9)	1/1 (5.6)			2/2 (5.6)
AEs leading to withdrawal			1/1 (5.9)				1/1 (2.8)
Most frequent AEs by PT (≥15% participants)							
Headache	2/2 (11.1)			8/6 (33.3)	6/4 (22.2)	2/2 (11.8)	18/10 (27.8)
Chromaturia					8/8 (44.4)		8/8 (22.2)
Fatigue	1/1 (5.6)			3/3 (16.7)	2/2 (11.1)	2/2 (11.8)	8/7 (19.4)
Catheter site irritation	1/1 (5.6)			4/3 (16.7)	2/2 (11.1)	3/3 (17.6)	10/6 (16.7)
Infusion site irritation	1/1 (5.6)		1/1 (5.9)	1/1 (5.6)		3/3 (17.6)	6/6 (16.7%)
Hot flush			2/2 (11.8)			4/4 (23.5)	6/6 (16.7)
FMGX-related AEs	1/1 (5.6)		3/3 (17.6)	15/5 (27.8)		18/8 (47.1)	37/12 (33.3)
FMGX-related AEs by SOC and PT^*a*^							
Nervous system disorders	1/1 (5.6)			8/5 (27.8)		5/4 (23.5)	14/7 (19.4)
Headache	1/1 (5.6)			5/4 (22.2)		1/1 (5.9)	7/6 (16.7)
Dizziness						2/2 (11.8)	2/2 (5.6)
Paraesthesia				2/2 (11.1)		1/1 (5.9)	3/2 (5.6)
Head discomfort				1/1 (5.6)		1/1 (5.9)	2/1 (2.8)
Gastrointestinal disorders				3/2 (11.1)		5/4 (23.5)	8/4 (11.1)
Nausea				2/2 (11.1)		4/4 (23.5)	6/4 (11.1)
Vomiting				1/1 (5.6)		1/1 (5.9)	2/1 (2.8)
Vascular disorders			2/2 (11.8)			4/4 (23.5)	6/6 (16.7)
Hot flush			2/2 (11.8)			4/4 (23.5)	6/6 (16.7)
General disorders and administration site conditions			1/1 (5.9)	1/1 (5.6)		2/2 (11.8)	4/4 (11.1)
Feeling hot			1/1 (5.9)	1/1 (5.6)		1/1 (5.9)	3/3 (8.3)
Chest pain						1/1 (5.9)	1/1 (2.8)
Skin and subcutaneous tissue disorders				2/1 (5.6)			2/1 (2.8)
Erythema				2/1 (5.6)			2/1 (2.8)
Metabolism and nutrition disorders				1/1 (5.6)			1/1 (2.8)
Decreased appetite				1/1 (5.6)			1/1 (2.8)
Musculoskeletal and connective tissue disorders						1/1 (5.9)	1/1 (2.8)
Back pain						1/1 (5.9)	1/1 (2.8)
Respiratory, thoracic, and mediastinal disorders						1/1 (5.9)	1/1 (2.8)
Dyspnea						1/1 (5.9)	1/1 (2.8)

^*a*^ AEs were coded using MedDRA version v22.1 (Participants were counted once, per preferred term, of multiple occurrences of a specific MedDRA term).

^*b*^ AE, adverse event; BID, twice a day; D, day; DDI, drug-drug interaction; E, number of AEs; FMGX, fosmanogepix; ITR, itraconazole; IV, intravenous; MedDRA, Medical Dictionary for Regulatory Activities; N, number of participants exposed; n, number of participants that experienced the AE; PO, oral; PT, preferred term; QD, once daily; RIF, rifampin; SOC, system organ class; %, number of participants (n) as a percentage of number of participants (N) per treatment.

^*c*^ Treatments: FMGX 500 mg: 500 mg FMGX IV (3-hour infusion) BID on Day 1. Time period covers Days 1 to 14, and Day 15 prior to the first ITR dose. ITR 200 mg: 200 mg ITR QD oral dosing on Days 15–17. Time period covers Days 15 (post-ITR dosing) to 17, and Day 18 (prior to FMGX morning infusion). FMGX + ITR: 500 mg FMGX IV (3-hour infusion) BID on Day 18 + 200 mg ITR QD oral dosing on Days 18–30. Time period covers Day 18 (post FMGX morning infusion) through follow-up (Day 38 ± 1). FMGX 1,000 mg: 1,000 mg FMGX IV (3-hour infusion) BID on Day 1. Time period covers Days 1 to 14, and Day 15 prior to the first RIF dose. RIF 600 mg: 600 mg RIF QD oral dosing on Days 15–23. Time period covers Days 15 (post-RIF dosing) to 23, and Day 24 (prior to FMGX morning infusion). FMGX + RIF: 1,000 mg FMGX IV (3-hour infusion) BID on Day 24 + 600 mg RIF QD oral dosing on Days 24–33. Time period covers Day 24 (post-FMGX morning infusion) through follow-up (Day 41 ± 1).

The majority (*n* = 186/188) of AEs were considered mild and two AEs [*n* = 2 (5.6%)] were considered moderate. These were elevated hepatic transaminases in one participant in cohort 1, occurring on the last day of itraconazole dosing (day 30, i.e., 12 days post last FMGX dose on day 18), considered related to itraconazole, and headache experienced by one participant in cohort 2 receiving FMGX 1000 mg only on Day 1, considered unrelated to any of the study drugs administered (Table 4).

Except for one participant (cohort 1) with clinically significant increase in hepatic transaminases, considered related to itraconazole (reported as a moderate AE, described above), no other clinically significant laboratory, vital signs, ECG, or physical examination findings were reported. In this participant, clinically significant increases in alanine transaminase [ALT; up to ~8× upper limit of normal (ULN)] and aspartate transaminase (AST; up to ~11× ULN) levels were observed on days 31 through day 70 and remained above normal limits until day 77 (AST) and day 84 (ALT), both returning to below ULN by day 91 (~7 to 8 weeks after last dose of itraconazole on day 30). A clinically significant increase in lactate dehydrogenase (LDH) levels was observed from day 31 to day 63 (up to ~2× ULN). The participant remained asymptomatic during the entire follow-up period and an ultrasound of the liver showed no abnormalities.

Among the most frequently reported AEs by system organ class (SOC; ≥20% participants), general disorders and administration site conditions were reported most frequently in both cohort 1 (post-FMGX + itraconazole administration) and cohort 2 (post-FMGX only and FMGX + rifampin administration). However, AEs in the nervous system, gastrointestinal, and renal and urinary disorders SOCs were reported more frequently in cohort 2 than in cohort 1 (Table S2). Among all treatment groups in both cohorts, the most frequently reported AEs by preferred term (PT; ≥15% participants) were headache, chromaturia, fatigue, catheter site irritation, infusion site irritation, and hot flush (Table 4). The percentage of participants reporting these was higher in cohort 2 vs cohort 1. Chromaturia, a known AE of rifampin use, was reported only during rifampin administration.

## DISCUSSION

An increasing number of fungal species (at least 300 to date) are known to cause infections in humans (14). FMGX, a novel antifungal, has demonstrated a favorable PK and safety profile in multiple phase 1 clinical studies and is currently under development for the treatment of IFDs (5, 6). Immunocompromised individuals and those with co-morbidities who are already susceptible to IFDs are also at risk of DDIs due to the co-administration of multiple drugs. Itraconazole is a strong CYP3A4 inhibitor with a well-established safety profile (12). Rifampin is a potent inducer of multiple CYP enzymes, including CYP1A2, CYP2B6, CYP2C9, CYP2C19, and CYP3A4, among others (15). Both itraconazole and rifampin are recommended by the FDA for use in DDI studies (11, 12). This phase 1, open-label, fixed-sequence study in healthy participants was conducted to assess whether FMGX and MGX PK profiles are altered in the presence of the CYP3A4 inhibitor itraconazole (cohort 1) or pan-CYP inducer rifampin (cohort 2).

In both cohorts, FMGX was administered as an IV infusion at a dose of 500 mg (cohort 1) and 1,000 mg (cohort 2). Based on the terminal elimination half-life of approximately 78 hours for MGX, the washout period of a minimum of 14 days between the two FMGX dosing days in both cohorts was sufficient to minimize any carryover effects from the initial administration. Since maximum CYP3A4 inhibition (itraconazole) and CYP induction (rifampin) is observed a few days after dosing (~3 days for itraconazole and 9 days for rifampin) (11, 12), the second FMGX dose was administered accordingly in each cohort, to maintain stable maximal CYP inhibition (cohort 1) and induction (cohort 2) throughout the FMGX PK sampling period.

In cohort 1, to avoid possible toxicities due to potential increased MGX exposures resulting from CYP3A4 inhibition, the FMGX dose administered was 500 mg, lower than 600/800 mg (IV/oral), that is, the doses evaluated in phase 2 trials and to be assessed in phase 3 trials (6, 16). Co-administration of multiple doses of itraconazole with a single FMGX dose was found to have no effect on the C_max_, AUC_0–t_, and AUC_inf_ of MGX, indicating no effect of CYP3A4 inhibition on MGX exposure.

In cohort 2, to maintain MGX exposures equivalent to levels observed with the doses evaluated in phase 2 trials/to be assessed in phase 3 trials [600/800 mg (IV/oral)] (6, 16) after possible pan-CYP induction, FMGX was administered at a dose of 1,000 mg. Co-administration of multiple doses of rifampin with a single FMGX dose did not significantly affect the C_max_ of MGX but did result in a ~45% decrease in the extent of MGX exposure as measured by AUC_0-t_ and AUC_inf_. MGX half-life also decreased from 70.7 to 41.7 hours, due to increased clearance of MGX after CYP induction (3,403 vs 1,862 mL/h). Similar results were observed when rifampin was co-administered with triazole antifungals (17–19), decreasing their therapeutic effects by reducing plasma exposures.

Similar to the DDI study of rifampin and isavuconazole (17), most AEs in this study were mild (186 of 188). Both FMGX doses (500 and 1,000 mg), administered either alone or with itraconazole 200 mg or rifampin 600 mg, respectively, were found to be safe and well tolerated. All AEs were transient and were resolved/resolving at follow-up. No deaths or SAEs were reported, and one participant withdrew due to mild anxiety after receiving FMGX and itraconazole, considered unrelated to FMGX. Two moderate AEs were reported, both unrelated to FMGX. In this study, the number of AEs, percentage of participants experiencing AEs, and number of AEs possibly related to FMGX were higher in cohort 2 compared to cohort 1, likely due to the higher FMGX dose in cohort 2 (1,000 mg) vs cohort 1 (500 mg).

In summary, co-administration of FMGX with itraconazole (a strong CYP3A4 inhibitor) did not affect MGX exposures, suggesting that dose modifications of FMGX may not be required when administered in combination with other CYP3A4 inhibitors. Co-administration of FMGX with rifampin (a pan-CYP inducer) reduced MGX AUC by ~45%, suggesting that FMGX dose increases may be considered during co-administration with CYP inducers to maintain plasma exposures. However, due to the minimal effect of rifampin on MGX C_max_ (13,897 vs 12,112 ng/mL) in our study, dose increases may also result in increases in C_max_, and therefore, a higher probability of experiencing drug-related AEs or toxicities. Hence, adjustments in dose or dosing frequency may be considered based on modeling and simulation to provide matching exposures in patients on strong CYP inducers. In addition, in contrast to healthy participants in this study (results not confounded by changes in disease state and/or effects of other concomitant medications), differences in patient metabolism may affect drug exposures when co-administered with CYP inducers. Traditionally, itraconazole shows non-linear PK and has multiple metabolites with inhibition potential (12, 20). However, no effect of itraconazole on MGX exposures was observed in this study. The preliminary results from this study indicate that therapeutic drug monitoring may not be necessary during co-administration with CYP inhibitors; however, closer monitoring may be required during co-administration with strong CYP inducers like rifampin due to the potential for loss of efficacy. The findings of this study, along with efficacy and safety data, indicate that physiologically based PK (PBPK) simulations may be considered to adjust FMGX dose or dosing frequency to maintain exposures in patients receiving strong CYP inducers like rifampin.

## Data Availability

Upon request, and subject to review, Pfizer will provide the data that support the findings of this study. Subject to certain criteria, conditions, and exceptions, Pfizer may also provide access to the related individual de-identified participant data. See https://www.pfizer.com/science/clinical-trials/trial-data-and-results for more information.

## SUPPLEMENTAL MATERIAL

Supplemental tables aac.01650-23-s0001.docx Tables S1 and S2.

ASM does not own the copyrights to Supplemental Material that may be linked to, or accessed through, an article. The authors have granted ASM a non-exclusive, world-wide license to publish the Supplemental Material files. Please contact the corresponding author directly for reuse.
