# Supplementary material for: Phase 1 drug-drug interaction study to assess the effect of CYP3A4 inhibition and pan-CYP induction on the pharmacokinetics and safety of fosmanogepix in healthy participants
Source: Antimicrob Agents Chemother. 2024 May 17;68(6):e01650-23. doi: 10.1128/aac.01650-23 (PMC11620482; doi:10.1128/aac.01650-23)
Supplement: Supplemental tables — Tables S1 and S2. [file aac.01650-23-s0001.docx]

**Supplementary Tables:**

## **Table S1. Summary of PK Parameters for FMGX (PK Analysis Set)**

|  | **Cohort 1: DDI with ITR (n = 16)** | | **Cohort 2: DDI with RIF (n = 17)** | |
| --- | --- | --- | --- | --- |
| **PK Parameter*** | **FMGX only (500 mg BID IV;D1)** | **FMGX (500 mg BID IV;D18) + ITR (200 mg QD PO; D15-30)** | **FMGX only (1000 mg BID IV;D1)** | **FMGX (1000 mg BID IV;D24) + RIF (600 mg QD PO; D15-33)** |
| C_max_(ng/mL) | 1520 (18.6) | 1516 (15.1) | 2974 (24.2) | 3395 (39.7) |
| T_max_ (hr) | 10.5  (0.50 – 12.0) | 6.53  (0.50 – 12.0) | 9.50  (0.50 – 12.1) | 11.0  (0.50 – 12.0) |
| AUC_(0-23)_ (hr×ng/mL) | 9945 (16.5) | 9454 (18.2) | 18162 (22.3) | 21166 (27.6) |
| AUC_(0-t)_ (hr×ng/mL) | 9945 (16.5) | 9454 (18.2) | 18162 (22.3) | 21166 (27.6) |
| AUC_(inf)_ (hr×ng/mL) | 10191 (18.3)** | 9454 (18.2) | 18163 (22.3) | 21167 (27.6) |
| λz (1/hr) | 1.83 (14.4)** | 1.76 (31.0) | 1.59 (18.4) | 1.68 (21.2) |
| t½ (hr) | 0.38 (14.4)** | 0.39 (31.0) | 0.44 (18.4) | 0.41 (21.2) |
| CL (mL/hr) | 98128 (18.3)** | 105770 (18.2) | 110117 (22.3) | 94488 (27.6) |
| Vz (L) | 53.6 (22.5)** | 60.1 (33.7) | 69.4 (30.4) | 56.4 (29.8) |

*Geometric mean [geometric %CV] except for Tlag and Tmax for which the median [Range] is reported; **n=12

AUC_(0-23)_, area under the plasma concentration-time curve (AUC) from time 0 to 23 hours; AUC_(0-inf)_, AUC from time 0 to infinity; AUC_(0-t)_, AUC up to time t, where t= last point with concentrations above the lower limit of quantitation (LLOQ); BID, twice a day; C_max_, maximum observed plasma concentration; CL, clearance, calculated as dose/AUC_0-inf_; CV, coefficient of variation; D, day; DDI, drug-drug interaction; FMGX, fosmanogepix; hr, hour; ITR, itraconazole; IV, intravenous; PK, pharmacokinetic(s); PO, oral; QD, once daily; RIF, rifampin; T_max_, time to attain C_max_; λz, terminal phase rate constant after the last dosing on the day; t1/2, terminal elimination phase half-life after the last dosing on the day; Vz volume of distribution at terminal phase

## **Table S2. Summary of All AEs by SOC and PT (Safety Analysis Set)**

|  | **Cohort 1: DDI with ITR (n=18)** | | | **Cohort 2: DDI with RIF (n=18)** | | | **Total (N=36)** |
| --- | --- | --- | --- | --- | --- | --- | --- |
| **SOC and PT*** | **FMGX only (500 mg BID IV;D1) (n=18)  E/n (%)** | **ITR only (200 mg QD PO; D15-17) (n=17) E/n (%)** | **FMGX (500 mg BID IV;D18) + ITR (200 mg QD PO; D18-30) (n=17) E/n (%)** | **FMGX only (1000 mg BID IV;D1) (n=18) E/n (%)** | **RIF only (600 mg QD PO; D15-23) (n=18) E/n (%)** | **FMGX (1000 mg BID IV;D24) + RIF (600 mg QD PO; D24-33) (n=17) E/n (%)** | **Overall**  **E/n (%)** |
| Any AEs | 21/9 (50.0) | 2/1 (5.9) | 20/9 (52.9) | 49/14 (77.8) | 47/13 (72.2) | 49/14 (82.4) | 188/30 (83.3) |
| **General disorders and administration site conditions** | 11/6 (33.3) |  | 9/7 (41.2) | 11/9 (50.0) | 7/4 (22.2) | 17/8 (47.1) | 55/22 (61.1) |
| Fatigue | 1/1 (5.6) |  |  | 3/3 (16.7) | 2/2 (11.1) | 2/2 (11.8) | 8/7 (19.4) |
| Catheter Site Irritation | 1/1 (5.6) |  |  | 4/3 (16.7) | 2/2 (11.1) | 3/3 (17.6) | 10/6 (16.7) |
| Infusion Site Irritation | 1/1 (5.6) |  | 1/1 (5.9) | 1/1 (5.6) |  | 3/3 (17.6) | 6/6 (16.7) |
| Catheter Site Haematoma | 6/3 (16.7) |  | 2/2 (11.8) |  | 2/1 (5.6) |  | 10/5 (13.9) |
| Catheter Site Pain | 1/1 (5.6) |  |  |  |  | 3/3 (17.6) | 4/4 (11.1) |
| Chest Pain |  |  | 2/2 (11.8) |  |  | 1/1 (5.9) | 3/3 (8.3) |
| Feeling Hot |  |  | 1/1 (5.9) | 1/1 (5.6) |  | 1/1 (5.9) | 3/3 (8.3) |
| Infusion Site Haematoma |  |  |  | 1/1 (5.6) | 1/1 (5.6) | 1/1 (5.9) | 3/3 (8.3) |
| Infusion Site Pain |  |  | 1/1 (5.9) |  |  |  | 1/1 (2.8) |
| Injection Site Irritation |  |  |  |  |  | 1/1 (5.9) | 1/1 (2.8) |
| Injection Site Pruritus |  |  | 1/1 (5.9) |  |  |  | 1/1 (2.8) |
| Medical Device Site Reaction | 1/1 (5.6) |  |  |  |  |  | 1/1 (2.8) |
| Pain |  |  |  |  |  | 1/1 (5.9) | 1/1 (2.8) |
| Puncture Site Erythema |  |  | 1/1 (5.9) |  |  |  | 1/1 (2.8) |
| Thirst |  |  |  | 1/1 (5.6) |  |  | 1/1 (2.8) |
| Vessel Puncture Site Haematoma |  |  |  |  |  | 1/1 (5.9) | 1/1 (2.8) |
| **Nervous system disorders** | 2/2 (11.1) |  | 1/1 (5.9) | 15/11 (61.1) | 11/7 (38.9) | 8/5 (29.4) | 37/16 (44.4) |
| Headache | 2/2 (11.1) |  |  | 8/6 (33.3) | 6/4 (22.2) | 2/2 (11.8) | 18/10 (27.8) |
| Somnolence |  |  |  | 3/3 (16.7) | 2/2 (11.1) |  | 5/4 (11.1) |
| Dizziness |  |  |  | 1/1 (5.6) | 1/1 (5.6) | 2/2 (11.8) | 4/3 (8.3) |
| Paraesthesia |  |  |  | 2/2 (11.1) | 1/1 (5.6) | 1/1 (5.9) | 4/3 (8.3) |
| Dysgeusia |  |  |  |  | 1/1 (5.6) | 1/1 (5.9) | 2/2 (5.6) |
| Head Discomfort |  |  |  | 1/1 (5.6) |  | 2/1 (5.9) | 3/1 (2.8) |
| Hypoaesthesia |  |  | 1/1 (5.9) |  |  |  | 1/1 (2.8) |
| **Gastrointestinal disorders** | 1/1 (5.6) | 2/1 (5.9) | 1/1 (5.9) | 9/5 (27.8) | 8/6 (33.3) | 9/5 (29.4) | 30/10 (27.8) |
| Nausea |  |  |  | 3/3 (16.7) | 2/2 (11.1) | 6/5 (29.4) | 11/5 (13.9) |
| Abdominal Pain |  | 1/1 (5.9) |  |  | 2/2 (11.1) | 1/1 (5.9) | 4/3 (8.3) |
| Faeces Soft |  |  | 1/1 (5.9) | 1/1 (5.6) | 1/1 (5.6) |  | 3/3 (8.3) |
| Diarrhoea | 1/1 (5.6) | 1/1 (5.9) |  |  |  |  | 2/2 (5.6) |
| Abdominal Discomfort |  |  |  | 1/1 (5.6) |  |  | 1/1 (2.8) |
| Abdominal Distension |  |  |  | 1/1 (5.6) |  |  | 1/1 (2.8) |
| Abdominal Pain Upper |  |  |  |  | 1/1 (5.6) |  | 1/1 (2.8) |
| Constipation |  |  |  | 1/1 (5.6) |  |  | 1/1 (2.8) |
| Frequent Bowel  Movements |  |  |  |  | 1/1 (5.6) |  | 1/1 (2.8) |
| Oral Pain |  |  |  |  |  | 1/1 (5.9) | 1/1 (2.8) |
| Regurgitation |  |  |  |  | 1/1 (5.6) |  | 1/1 (2.8) |
| Stomatitis |  |  |  | 1/1 (5.6) |  |  | 1/1 (2.8) |
| Vomiting |  |  |  | 1/1 (5.6) |  | 1/1 (5.9) | 2/1 (2.8) |
| **Respiratory, thoracic and mediastinal disorders** | 4/3 (16.7) |  | 2/2 (11.8) | 3/2 (11.1) |  | 4/3 (17.6) | 13/10 (27.8) |
| Dyspnoea |  |  | 2/2 (11.8) |  |  | 1/1 (5.9) | 3/3 (8.3) |
| Cough |  |  |  | 2/2 (11.1) |  |  | 2/2 (5.6) |
| Epistaxis | 1/1 (5.6) |  |  |  |  | 2/1 (5.9) | 3/2 (5.6) |
| Nasal Congestion |  |  |  | 1/1 (5.6) |  |  | 1/1 (2.8) |
| Oropharyngeal Pain | 1/1 (5.6) |  |  |  |  |  | 1/1 (2.8) |
| Pharyngeal Swelling | 1/1 (5.6) |  |  |  |  |  | 1/1 (2.8) |
| Rhinorrhoea | 1/1 (5.6) |  |  |  |  |  | 1/1 (2.8) |
| Sputum Increased |  |  |  |  |  | 1/1 (5.9) | 1/1 (2.8) |
| **Renal and urinary disorders** |  |  |  | 1/1 (5.6) | 9/8 (44.4) |  | 10/8 (22.2) |
| Chromaturia |  |  |  |  | 8/8 (44.4) |  | 8/8 (22.2) |
| Pollakiuria |  |  |  | 1/1 (5.6) | 1/1 (5.6) |  | 2/1 (2.8) |
| **Skin and subcutaneous**  **tissue disorders** | 1/1 (5.6) |  | 2/2 (11.8) | 3/2 (11.1) |  | 4/4 (23.5) | 10/7 (19.4) |
| Skin Irritation | 1/1 (5.6) |  | 1/1 (5.9) | 1/1 (5.6) |  | 2/2 (11.8) | 5/5 (13.9) |
| Erythema |  |  |  | 2/1 (5.6) |  | 1/1 (5.9) | 3/2 (5.6) |
| Pruritus |  |  |  |  |  | 1/1 (5.9) | 1/1 (2.8) |
| Yellow Skin |  |  | 1/1 (5.9) |  |  |  | 1/1 (2.8) |
| **Vascular disorders** |  |  | 2/2 (11.8) | 1/1 (5.6) |  | 4/4 (23.5) | 7/7 (19.4) |
| Hot Flush |  |  | 2/2 (11.8) |  |  | 4/4 (23.5) | 6/6 (16.7) |
| Haematoma |  |  |  | 1/1 (5.6) |  |  | 1/1 (2.8) |
| **Musculoskeletal and connective tissue disorders** | 2/2 (11.1) |  |  | 1/1 (5.6) | 1/1 (5.6) | 2/2 (11.8) | 6/6 (16.7) |
| Back Pain |  |  |  | 1/1 (5.6) |  | 2/2 (11.8) | 3/3 (8.3) |
| Myalgia | 1/1 (5.6) |  |  |  | 1/1 (5.6) |  | 2/2 (5.6) |
| Limb Discomfort | 1/1 (5.6) |  |  |  |  |  | 1/1 (2.8) |
| **Psychiatric disorders** |  |  | 1/1 (5.9) | 1/1 (5.6) | 4/3 (16.7) |  | 6/4 (11.1) |
| Anxiety |  |  | 1/1 (5.9) |  |  |  | 1/1 (2.8) |
| Disorientation |  |  |  | 1/1 (5.6) |  |  | 1/1 (2.8) |
| Insomnia |  |  |  |  | 2/1 (5.6) |  | 2/1 (2.8) |
| Irritability |  |  |  |  | 1/1 (5.6) |  | 1/1 (2.8) |
| Nightmare |  |  |  |  | 1/1 (5.6) |  | 1/1 (2.8) |
| **Eye disorders** |  |  |  | 1/1 (5.6) | 2/1 (5.6) | 1/1 (5.9) | 4/2 (5.6) |
| Asthenopia |  |  |  | 1/1 (5.6) |  |  | 1/1 (2.8) |
| Eye Irritation |  |  |  |  |  | 1/1 (5.9) | 1/1 (2.8) |
| Ocular Hyperaemia |  |  |  |  | 2/1 (5.6) |  | 2/1 (2.8) |
| **Metabolism and nutrition disorders** |  |  |  | 1/1 (5.6) | 3/3 (16.7) |  | 4/4 (11.1) |
| Decreased Appetite |  |  |  | 1/1 (5.6) | 3/3 (16.7) |  | 4/4 (11.1) |
| **Infections and infestations** |  |  |  | 1/1 (5.6) | 2/2 (11.1) |  | 3/3 (8.3) |
| Nasopharyngitis |  |  |  | 1/1 (5.6) | 2/2 (11.1) |  | 3/3 (8.3) |
| **Ear and labyrinth disorders** |  |  | 1/1 (5.9) |  |  |  | 1/1 (2.8) |
| Tinnitus |  |  | 1/1 (5.9) |  |  |  | 1/1 (2.8) |
| **Investigations** |  |  | 1/1 (5.9) |  |  |  | 1/1 (2.8) |
| Hepatic Enzyme Increased |  |  | 1/1 (5.9) |  |  |  | 1/1 (2.8) |
| **Reproductive system and breast disorders** |  |  |  | 1/1 (5.6) |  |  | 1/1 (2.8) |
| Vulvovaginal Dryness |  |  |  | 1/1 (5.6) |  |  | 1/1 (2.8) |

*AEs were coded using MedDRA version v22.1 (Participants were counted once, per preferred term, of multiple occurrences of a specific MedDRA term).

AE, adverse event; BID, twice a day; D, day; DDI, drug-drug interaction; E, number of AEs; FMGX, fosmanogepix; ITR, itraconazole; IV, intravenous; MedDRA, Medical Dictionary for Regulatory Activities; N, number of participants exposed; n, number of participants that experienced the AE; PO, oral; PT, preferred term; QD, once daily; RIF, rifampin; SOC, system organ class; %, number of participants (n) as a percentage of number of participants (N) per treatment.

Treatments:

FMGX 500 mg: 500 mg FMGX IV (3-hour infusion) BID on Day 1. Time period covers Days 1 to 14, and Day 15 prior to first ITR dose.

ITR 200 mg: 200 mg ITR QD oral dosing on Days 15-17. Time period covers Days 15 (post ITR dosing) to 17, and Day 18 (prior to FMGX morning infusion).

FMGX + ITR: 500 mg FMGX IV (3-hour infusion) BID on Day 18 + 200 mg ITR QD oral dosing on Days 18-30. Time period covers Day 18 (post FMGX morning infusion) through follow-up (Day 38±1).

FMGX 1000 mg: 1000 mg FMGX IV (3-hour infusion) BID on Day 1. Time period covers Days 1 to 14, and Day 15 prior to first RIF dose.

RIF 600 mg: 600 mg RIF QD oral dosing on Days 15-23. Time period covers Days 15 (post RIF dosing) to 23, and Day 24 (prior to FMGX morning infusion).

FMGX + RIF: 1000 mg FMGX IV (3-hour infusion) BID on Day 24 + 600 mg RIF QD oral dosing on Days 24-33. Time period covers Day 24 (post FMGX morning infusion) through follow-up (Day 41±1)
